# Gender and locality differences in tobacco prevalence among adult Bangladeshis

**DOI:** 10.1136/tc.2008.028142

**Published:** 2009-08-13

**Authors:** M S Flora, C G N Mascie-Taylor, M Rahman

**Affiliations:** 1 NIPSOM, Dhaka, Bangladesh; 2 Department of Biological Anthropology, University of Cambridge, UK; 3 IEDCR, Dhaka, Bangladesh

## Abstract

**Objectives:**

To determine the extent of all forms of tobacco usage in adult Bangladeshis in relation to gender and locality.

**Methods:**

Three annual urban and rural cross-sectional surveys were carried out between 2001 and 2003 involving a total of 35 446 adults, of whom 54.3% were female and 51.0% were rural dwellers. Data were collected through interview using a structured questionnaire.

**Results:**

The overall prevalences of smoking, chewing tobacco and gul usage were 20.5%, 20.6% and 1.8%, respectively. Current smoking and gul usage were significantly higher in males (42.2% and 2.2%, respectively) than females (2.3% and 1.5%, respectively) while chewing tobacco was more common in females (21.6%) than males (19.4%). No significant urban-rural difference was observed in smoking rate after adjusting for sociodemographic variables, while chewing tobacco was 1.5 times more likely to occur in rural residents and gul usage was 3.6 times more likely to occur in urban residents. On average a smoker consumed 9.3 sticks a day with males and rural residents smoking more.

**Conclusions:**

Nearly a third of the population in Bangladesh use some form of tobacco. There are marked urban-rural and male-female differences. This difference is mainly accounted for by the higher prevalence of chewing tobacco in rural areas, rural female tobacco usage is close to double than the urban rate. Smoking rates were low in Bangladeshi females, more so in urban than rural areas. The tobacco awareness programme in Bangladesh might require putting emphasis on smokeless tobacco as well as smoking.

Tobacco is a major avoidable cause of illness and premature death in low-income countries.^1^ There were an estimated 4.83 million premature deaths attributable to smoking in 2000, 12% of the total global adult mortality.^2^ If current smoking patterns continue, it will cause some 10 million deaths each year by 2020.^3^ In South Asia, chewing tobacco is a major cause of oral cancer.^4^

Tobacco use is not exclusively, or even principally, a problem in developed countries; it is rapidly becoming a global pandemic, infiltrating even the poorest nations.^5^ About one in every three adults smokes and the majority are in developing countries (800 million) and most of them are male (700 million). The smoking epidemic is spreading from its original focus, among men in high-income countries to women in high-income countries and men in low-income and medium-income countries.^3^ Tobacco is used in a number of forms in South Asia.^6^ The prevalence of at least one form of tobacco daily in Bangladesh ranged between 33.4% and 41%^7^ ^8^; smoking rate varied between 21% to 25%^9^ ^10^ ^11^; the chewed tobacco rates were 17% in Pakistan,^12^ 21% in India^13^; and the prevalence of gul use in Bangladesh was 1.5%,^11^ whereas it was 25.7% in India.^14^ The distribution of tobacco consumption is not uniform; it is disproportionately higher in lower socioeconomic groups,^13^ poor, semi-skilled manual occupation groups, unemployed and poor educational achievers.^15^ In countries of South Asia traditional values do not permit smoking by women, but there is no such taboo against using smokeless tobacco.^4^ The use of chewing tobacco, bidis (hand-rolled cigarettes) and cigarettes is widespread in Bangladesh and an estimated 70% of the tobacco produced is used for cigarettes and bidis, 20% is consumed as chewing tobacco and the remaining 10% is used in other forms of tobacco. From 1992 to 1996, annual per capita consumption of cigarettes increased by 33%.^6^

Data on tobacco usage are scant or lacking in many of the poorest nations. The Bangladeshi tobacco data, so far available, are based on small-scale studies. At country level, core public health functions such as health monitoring, health surveillance and public health research are needed. Monitoring and surveillance data in relation to tobacco use and its impacts is one of the main requirements. In relation to monitoring and surveillance, standardised estimates of smoking prevalence (that is, using the agreed standardised definitions of tobacco use) are needed to chart the progress of developing countries through the stages of the tobacco epidemic, and to determine specific strategies for intervention. Without these data, the extent and range of the impact of tobacco cannot be gauged. This study aimed to find out the magnitude of tobacco usage in Bangladesh with particular focus on variation by gender and locality.

## Methods

Three annual urban and rural cross-sectional studies were conducted between 2001 and 2003 with the aim of sampling about 5730 individuals from each area per year. The surveys were undertaken in an urban area, Mirpur, Dhaka City, the capital of Bangladesh and the rural sample was from Kaliganj Upazila (subdistrict), which is approximately 42 km from the capital. Mirpur and Kaliganj were selected purposely as both the areas represent general urban and rural populations of Bangladesh, respectively. The age distribution of this sample is similar to the adult Bangladeshi age pyramid found in the national survey carried out in 2003.^16^ The background characteristics of this sample appear to reflect the profile of the adult Bangladesh population.^16^ ^17^ These two areas were chosen to assess whether type, quantity and quality of tobacco products consumed by people differed markedly between rural and urban areas, independent of variation in socioeconomic background. A systematic sampling protocol was used based on the total adult population size in these two areas, and recruitment of every alternate household, which fulfilled the selection criteria, was planned. To ensure equal representation of both sexes at least one male and one female respondent from each household was included in the study. Overall, 89% and 77% of the invited households in rural and urban area, respectively, consented to participate in the surveys. Four teams comprising one male and one female interviewer, recruited from the local community, collected data from the households. Male household members were interviewed by male interviewers and female interviewers collected data from the female members. Before data collection, the interviewers were initially intensively trained in a 5-day programme on use of the questionnaire, data collection and measurements, selection of study participants and sampling before the first round of data collection. Every year there was a 4-day refresher course. During these training sessions all the data collectors were asked to measure the same set of individuals in order to determine the intra-observer and inter-observer variation. For assessing the intra-observer and inter-observer variation, technical error of measurements and coefficients of reliability were calculated three times, once each year. Coefficients of reliability values were higher than the threshold given by Ulijaszek and Kerr,^18^ and so were considered acceptable and, therefore, were not incorporated into the statistical analyses.

A pretested semi-structured questionnaire printed in Bangla was used for data collection. Verbal consent was obtained from every respondent and interviews were held in private. Ethical clearance was obtained from the institutional ethics committee.

The sociodemographic variables collected were age, sex, marital status, educational attainment, religion and main occupation. The tobacco usage variables provided information about smoking (current, past and occasional) and smokeless tobacco (chewing tobacco and gul). Current smokers were defined as those who smoked daily at the time of the data collection; past smokers those who had stopped smoking before the data collection period but used to smoke daily previously; and occasional smokers were those who smoked from time to time. The current smokers provided details on the different forms of smoking used, including cigarette and bidi (hand-rolled cigarettes that contain unprocessed tobacco) and others (include water pipes/hukkah) as well as on the numbers smoked per day. Tobacco is chewed as part of mixture with betel nut and gul is a moist, ground tobacco product placed between the cheek and gum.

The analyses were carried out primarily using the SPSS version 14.0. Data were weighted to account for the age distribution, gender and locality stratification. Statistical tests used to determine the association between exposure and outcome variables included χ^2^ test. A result was considered significant at a p value <0.05 but given the large sample sizes a more stringent cut-off of p<0.01, or less, was usually used. Effects of exposure variables were also assessed after adjusting for other variables by multivariate analyses. In addition to p value, 95% confidence intervals of different estimates were also given to show the range of values of the test statistic.

## Results

### Background characteristics

This study presents information on 35 446 individuals aged between 18 and 90 years with an overall geometric mean age of 33 (SD 15) years. More females were recruited overall (54.3%), but the sex ratios were very similar in each locality and in each year and 51% of respondents lived in rural area. Table 1 shows the variation in sociodemographic variables in relation to gender and locality. Females, especially, urban, were more often found in the younger age groups than males. The breakdown by age groups revealed higher male percentages in the 50 years and above groups. The vast majority of the respondents were Muslims (93.1%) and married (78.3%). Non-Muslim respondents, especially Hindus, were found more often in rural areas although no sex difference was found. There were more unmarried respondents in urban areas while a much higher percentage of women were either widowed or divorced. Males and urban respondents were generally better educated than females as well as those from rural areas. Excluding the non-paid, the main occupation was farming in rural areas, and service and business in urban areas. As would be expected occupation showed marked gender differences with four out of five females in non-paid (housework) work compared with about one in 12 males.

**Table 1:** Background characteristics of the study sample

Characteristics	Rural			Urban			Overall		
	Male	Female	Total	Male	Female	Total	Male	Female	Total
	No (%)	No (%)	No (%)	No (%)	No (%)	No (%)	No (%)	No (%)	No (%)
**Age group (years)**									
<20	780 (9.5)	995 (10.1)	1775 (9.8)	769 (9.7)	1243 (13.2)	2012 (11.6)	1549 (9.6)	2238 (11.6)	3787 (10.7)
20–29	1595 (19.4)	2847 (28.9)	4442 (24.6)	2604 (32.7)	3978 (42.3)	6582 (37.9)	4200 (25.9)	6824 (35.5)	11024 (31.1)
30–39	1795 (21.8)	2082 (21.1)	3877 (21.4)	2177 (27.3)	1804 (19.2)	3981 (22.9)	3972 (24.5)	3886 (20.2)	7858 (22.2)
40–49	1381 (16.8)	1876 (19.0)	3257 (18.0)	1137 (14.3)	1500 (16.0)	2637 (15.2)	2518 (15.5)	3376 (17.5)	5894 (16.6)
50–59	999 (12.1)	1184 (12.0)	2183 (12.1)	761 (9.6)	564 (6.0)	1325 (7.6)	1760 (10.9)	1748 (9.1)	3508 (9.9)
60–69	849 (10.3)	609 (6.2)	1458 (8.1)	405 (5.1)	248 (2.6)	653 (3.8)	1254 (7.8)	857 (4.4)	2111 (6.0)
70+	830 (10.1)	258 (2.6)	1088 (6.0)	114 (1.4)	62 (0.7)	176 (1.0)	944 (5.8)	320 (1.7)	1264 (3.5)
**Religion**									
Islam	7233 (87.9)	8661 (87.9)	15894 (87.9)	7836 (98.4)	9271 (98.7)	17107 (98.5)	15070 (93.1)	17931 (93.2)	33001 (93.1)
Hinduism	804 (9.8)	946 (9.6)	1750 (9.7)	94 (1.2)	104 (1.1)	198 (1.1)	898 (5.5)	1050 (5.4)	1948 (5.5)
Christianity	191 (2.3)	244 (2.5)	435 (2.4)	34 (0.4)	22 (0.2)	56 (0.4)	225 (1.4)	266 (1.4)	491 (1.4)
**Marital status**									
Married	6508 (79.1)	8462 (85.9)	14970 (82.8)	5686 (71.4)	7096 (75.5)	12782 (73.6)	12195 (75.3)	15557 (80.8)	27752 (78.3)
Unmarried	1627 (19.8)	597 (6.1)	2224 (12.3)	2260 (28.4)	1655 (17.6)	3915 (22.5)	3887 (24.0)	2252 (11.7)	6139 (17.3)
Widow/divorcees	94 (1.1)	792 (8.0)	886 (4.9)	21 (0.3)	647 (6.9)	668 (3.9)	115 (0.7)	1439 (7.5)	1554 (4.4)
**Educational status**									
No schooling	3147 (38.3)	4659 (47.3)	7806 (43.2)	826 (10.4)	1777 (18.9)	2603 (15.0)	3973 (24.6)	6436 (33.4)	10409 (29.4)
1–5 years of schooling	1954 (23.8)	2150 (21.8)	4104 (22.7)	1187 (14.9)	2357 (25.1)	3544 (20.4)	3141 (19.4)	4507 (23.4)	7648 (21.6)
6–10 years of schooling	2376 (28.9)	2768 (28.1)	5144 (28.5)	3020 (37.9)	3622 (38.6)	6642 (38.3)	5396 (33.3)	6390 (33.3)	11786 (33.3)
Higher secondary+	740 (9.0)	271 (2.8)	1011 (5.6)	2930 (36.8)	1638 (17.4)	4568 (26.3)	3671 (22.7)	1908 (9.9)	5579 (15.70)
**Occupation**									
Non-paid	537 (6.5)	9415 (97.0)	9952 (55.6)	789 (10.4)	6607 (72.4)	7396 (44.3)	1326 (8.4)	16022 (85.1)	17348 (50.1)
Students	345 (4.2)	229 (2.4)	574 (3.2)	812 (10.7)	789 (8.6)	1601 (9.6)	1157 (7.3)	1018 (5.4)	2175 (6.3)
Manual labourer	393 (4.8)	2 (0.02)	395 (2.2)	407 (5.4)	51 (0.6)	458 (2.7)	800 (5.1)	53 (0.3)	853 (2.5)
Farmer	4171 (50.8)	7 (0.1)	4178 (23.3)	26 (0.3)	3 (0.03)	29 (0.2)	4197 (26.6)	10 (0.1)	4207 (12.1)
Skilled labourer	401 (4.9)	5 (0.1)	406 (2.30	534 (7.1)	256 (2.8)	790 (4.7)	935 (5.9)	261 (1.4)	1196 (3.5)
Business	1484 (18.1)	11 (0.1)	1495 (8.3)	1972 (26.0)	263 (2.9)	2235 (13.4)	3456 (21.9)	274 (1.5)	3730 (10.8)
Service/professionals	875 (10.7)	35 (0.4)	910 (5.1)	3034 (40.1)	1156 (12.7)	4190 (25.1)	3909 (24.8)	1191 (6.3)	5100 (14.7)

### Prevalence of tobacco

The prevalence of any tobacco use was 39.4%, 36.4% and 36.6% in the years 2001, 2002 and 2003, respectively. A steady prevalence of smoking was observed in the three cross-sectional surveys (20.7% in 2001, 19.9% in 2002 and 21.0% in 2003). As tobacco prevalence did not show drastic difference in three surveys, for identification of locality and gender differences and other subsequent analyses total samples were considered. However, the effect of the surveys was removed during multivariate analyses.

There were significant age group differences between males and females in both rural and urban samples, so in order to compare between genders and by locality, sequential binary logistic regression was used to remove gender and locality effects where relevant, as well as other socioeconomic factors (marital status, religion, education, occupation and age groups).

Overall, 37.5% of this sample were tobacco users but there were more rural (43.9%) compared with urban users (30.9%) and males were nearly twice as likely to use tobacco compared with females (54.3% versus 23.4%, respectively, table 2). Of the total respondents 20.5% were current smokers, 8.1% indicated that they had stopped smoking and another 4.5% of the sample reported that they were occasional smokers. The percentage of current and past smokers was higher among rural than urban residents, whereas urban residents smoked occasionally in markedly higher proportions than their counterparts. There was a very strong gender difference with about 20 times more males currently smoking than females and the difference was higher in rural areas. The overall prevalence of chewing tobacco was 20.6% and rural residents were twice as likely to chew as urban residents and females chewed tobacco more than males. Overall, the prevalence of gul use was 1.8%. In urban areas there were more than double the percentage of users than in the rural areas and men used gul more than women. Dual tobacco (both smoking and smokeless tobacco) were used by 4.4% of the sample with higher prevalence in males and rural residents.

**Table 2:** Prevalence of tobacco

Tobacco	Rural			Urban			Overall		
	Male	Female	Total	Male	Female	Total	Male	Female	Total
	p Value (95% CI)	p Value (95% CI)	p Value (95% CI)	p Value (95% CI)	p Value (95% CI)	p Value (95% CI)	p Value (95% CI)	p Value (95% CI)	p Value (95% CI)
Any tobacco	60.7 (59.6 to 61.8)	29.8 (28.9 to 30.7)	43.9 (43.2 to 44.6)	47.6 (46.5 to 48.7)	16.7 (15.9 to 17.5)	30.9 (30.2 to 31.6)	54.3 (53.5 to 55.1)	23.4 (22.8 to 24.0)	37.5 (37.0 to 38.0)
Current smoking	43.2 (42.1 to 44.3)	4.2 (3.8 to 4.6)	21.9 (21.3 to 22.5)	41.1 (40.0 to 42.2)	0.3 (0.2 to 0.4)	19.1 (18.5 to 19.7)	42.2 (41.4 to 43.0)	2.3 (2.1 to 2.5)	20.5 (20.1 to 20.9)
Past smoking	16.2 (15.4 to 17.0)	6.9 (6.4 to 7.4)	11.1 (10.6 to 11.6)	9.9 (9.2 to 10.6)	0.6 (0.4 to 0.8)	4.9 (4.6 to 5.2)	13.1 (12.6 to 13.6)	3.8 (3.5 to 4.1)	8.1 (7.8 to 8.4)
Occasional smoking	1.1 (0.9 to 1.3)	0.2 (0.1 to 0.3)	0.6 (0.5 to 0.7)	17.4 (16.6 to 18.2)	1.1 (0.9 to 1.3)	8.6 (8.2 to 9.0)	9.1 (8.7 to 9.5)	0.7 (0.6 to 0.8)	4.5 (4.3 to 4.7)
Chewing tobacco	28.8 (27.8 to 29.8)	27.1 (26.2 to 28.0)	27.9 (27.2 to 28.6)	9.7 (9.0 to 10.4)	15.9 (15.2 to 16.6)	13.1 (12.6 to 13.6)	19.4 (18.8 to 20.0)	21.6 (21.0 to 22.2)	20.6 (20.2 to 21.0)
Gul	1.7 (1.4 to 2.0)	0.7 (0.5 to 0.9)	1.1 (0.9 to 1.3)	2.8 (2.4 to 3.2)	2.4 (2.1 to 2.7)	2.6 (2.4 to 2.8)	2.2 (2.0 to 2.4)	1.5 (1.3 to 1.7)	1.8 (1.7 to 1.9)
Dual tobacco use	12.1 (11.4 to 12.8)	1.7 (1.4 to 2.0)	6.4 (6.0 to 6.8)	4.8 (4.3 to 5.3)	0.2 (0.1 to 0.3)	2.3 (2.1 to 2.5)	8.5 (8.1 to 8.9)	1.0 (0.9 to 1.1)	4.4 (4.2 to 4.6)

Sequential binary logistic regression analyses were used to test whether gender and locality effects on tobacco usage remained significant after correcting for the other sociodemographic variables. Tables 3 and 4 present the findings of these analyses in which all of the sociodemographic variables were entered into the first block and locality or gender was entered into the second block.

**Table 3:** Influence of residential status on tobacco usage after adjusting for other sociodemographic variables (values shown for urban residents in reference to rural residents)

Tobacco usage	p Value	Odds ratio (95% CI)
Any tobacco	0.02	0.93 (0.87 to 0.98)
Current smoking	NS	0.92 (0.85 to 1.00)
Past smoking	<<0.001	0.50 (0.45 to 0.56)
Occasional smoking	<<<<0.001	15.17 (11.79 to 19.51)
Chewing tobacco	<<<<0.001	0.68 (0.63 to 0.73)
Gul	<<<<0.001	3.51 (2.83 to 4.36)
Dual tobacco	<<<<0.001	0.52 (0.45 to 0.59)

**Table 4:** Influence of gender on tobacco usage after adjusting for other sociodemographic variables (values shows for females in reference to males)

Tobacco usage	p Value	Odds ratio (95% CI)
Any tobacco	<<<<0.001	0.15 (0.13 to 0.17)
Current smoking	<<<<0.001	0.02 (0.02 to 0.03)
Past smoking	<<<<0.001	0.13 (0.12 to 0.14)
Occasional smoking	<<<<0.001	0.03 (0.02 to 0.03)
Chewing tobacco	<0.001	1.34 (1.17 to 1.53)
Gul	0.002	0.62 (0.45 to 0.85)
Dual tobacco	<<0.001	0.11 (0.08 to 0.15)

The rural/urban difference in any form of tobacco use and current smoking could mainly be accounted for by the other socioeconomic variables. Past smoking and chewing tobacco and dual tobacco use were found to be less common in urban areas while urban residents were more likely to smoke occasionally and use gul. The strong gender effect for smoking and other tobacco use remained after adjustment for the other sociodemographic variables (table 4). Females were less likely to smoke or use gul or use dual tobacco but more likely to chew tobacco.

All the current smokers in this sample were questioned about what they smoked and the majority (71.4%) reported cigarettes, while others smoked mainly bidi or hukkah. The types differed according to gender and locality (table 5).

**Table 5:** Bidi and cigarette smoking in relation to the locality and gender

Variables	Unadjusted		Adjusted for other sociodemographic variables	
	p Value	Odds ratio (95% CI)	p Value	Odds ratio (95% CI )
Area			<<0.001	
Rural*				
Urban	<<<<0.001	0.13 (0.11 to 0.15)		0.450 (0.372 to 0.543)
Sex			<<0.001	
Male*				
Female	<<<<0.001	34.83 (24.66 to 49.19)		12.315 (7.626 to 19.884)

*Reference group.

About one in 10 urban smokers, compared to about half of the rural residents smoked bidi and other forms of tobacco. Three-quarters of males smoked cigarettes compared with only one in 12 females; whereas females tended to smoke bidi and other types of tobacco (91.9%). Odds ratio shown in table 5 suggested that the urban residents were less likely and the females were more likely to smoke bidi and other types of tobacco. After removing the effects of the other sociodemographic variables by sequential binary logistic regression analysis females were about 12.3 times more likely to consume bidi and other forms of tobacco than males, while urban residents were less likely to use bidi than rural residents.

On average, 9.30 sticks (geometric mean) were smoked per day but considerable variation was observed in relation to the sociodemographic variables (table 6). Rural residents and males smoked, on average, more sticks/day. Multiple regression analysis showed that there was less frequent smoking in urban dwellers, and females. On average, bidi users smoked three sticks more than the cigarette smokers.

**Table 6:** Frequency of smoking per day in relation to locality and gender

Variables	Mean† (95% CI)	Adjusted regression coefficient (95% CI)
Area		
Rural*	10.69 (10.41 to 10.97)	
Urban	7.87 (7.63 to 8.11)	−0.143 (−0.157 to −0.129)
Sex		
Male*	9.60 (9.40 to 9.80)	
Female	5.76 (5.30 to 6.22)	−0.313 (−0.345 to −0.281)
Type of smoking		
Cigarette*	8.08 (7.89 to 8.27)	
Bidi and others	13.24 (12.82 to 13.66)	0.319 (0.305 to 0.333)

*Reference group; †geometric mean, adjusted for other sociodemographic variables.

The smokers were categorised into heavy (>20 sticks per day), moderate (10–20 sticks) and light (<10 sticks per day) smokers. Heavy smoking was more common among males than females (23.4% vs 5.4%), rural than urban residents (29.0% vs 15.2%) and bidi than cigarette smokers (43.0% vs 14.6%). After correcting for the sociodemographic variables the associations of gender, locality and type of smoking with level of smoking remained significant. The odds ratio suggested that in relation to light smoking, heavy smoking was 5.5 times (OR 5.51; 95% CI, 4.46 to 6.66) and moderate smoking 1.3 times (OR 1.26; 95% CI, 1.05 to 1.51) more likely to occur among the bidi smokers than the cigarette smokers. Likewise, both heavy and moderate smoking were more likely among rural (heavy smoking OR, 2.60; 95% CI, 2.16 to 3.12 and moderate smoking OR, 2.40; 95% CI, 2.06 to 2.79) and male residents (heavy smoking OR, 28.57; 95% CI, 2.16 to 47.62 and moderate smoking OR, 15.15; 95% CI, 9.62 to 23.81) than their counterparts.

## Discussion

Tobacco usage is thought to be a long-standing problem in Bangladesh, although until now no large scale study has been undertaken. The present study covering both urban and rural areas has been undertaken to answer some key questions about the extent of all forms of tobacco usage.

This study analysed data on 16 196 males and 19 250 females ⩾18 years of age with almost equal representation from Kaliganj (rural 51%) and Mirpur (urban 49%) areas. Although the aim was to obtain equal representation of gender and locality, more women were recruited (male:female ratio 0.84:1) which was similar in urban and rural areas. The female excess is primarily due to data collection during the daytime, when male members of the household were generally less available. It has already been mentioned in the methods section that the background characteristics of this sample appear to reflect the profile of the adult Bangladesh population.^16^ ^17^

Overall, 37.5% of this sample used at least one form of tobacco daily which is in keeping with other Bangladeshi studies where the prevalence ranged between 33.4% and 41%.^7^ ^8^ Rahman (2003) reported that tobacco prevalence has increased in Bangladesh from 37% to 41% in the past decade.^7^ However, the results from the current study do not indicate any increase in prevalence albeit over this short three-year time span.

Overall, 20.5% were current smokers, 8% had stopped smoking and 4.5% were occasional smokers. This current smoking rate is lower than the rates reported by the Bangladesh Bureau of Statistics (BBS) in 1996^9^ and 1999^10^ (25.2% and 23.1%, respectively) but similar to the rate (21.8%) found by Rahman *et al*.^11^ Rahman *et al* also reported a lower rate (4.9%) of past smoking than the current study but older groups were not included in that study, which might account for the difference.^11^

Overall, 20.6% of the current sample chewed tobacco, which is in broad agreement with rates in Pakistan (17%)^12^ and India (21%),^13^ although Indian prevalence rates vary between states, from 21.8% in Nagpur^19^ and 26.6% in Kerala^4^ to 56.7% in Mumbai.^14^ The prevalence of gul use was 1.8% in this sample, which is similar to a previous Bangladeshi study (1.5%),^11^ but much lower than in Mumbai, India where gul use was as high as 25.7%.^14^

Tobacco is used in a number of forms in South Asia.^6^ In the 1980s bidi smoking was common in Bangladesh, but since then smokers generally prefer cigarettes and in this sample 71.4% smoked cigarettes. In the study of Rahman *et al* all the sample smoked cigarettes.^11^ The average number of sticks (irrespective of type of smoking) smoked per day was 9.3, which is about one-third lower than the 12.6 sticks/day reported by Rahman *et al*.^11^ A study in Mumbai found that 4.6% were heavy smokers (>20 sticks),^14^ which is much lower than the present study (13.6%). No comparable Bangladeshi data were available. In addition to prevalence, consumption data are also needed to fully understand population exposure to the hazards of tobacco use.^20^ In this study the frequency of smoking varied with type of tobacco. Bidi users smoked significantly more frequently (on average, 13.2 sticks per day) than cigarette smokers (on average, 8.1 sticks per day) after adjusting for sociodemographic variables. The proportion of heavy smokers (>20 sticks/day) was about 10 times higher for bidi smokers (38.2%) than for cigarette smokers (3.8%). No other comparable Bangladeshi study exists but in India Gupta found similar percentages (34.9% of bidi and 4.6% of cigarette smokers were heavy smokers).^20^ Bidi smokers are exposing themselves to greater health hazards than cigarette smokers, as there are greater risks of developing cancers of the oral cavity, oesophagus, stomach, larynx, lungs and some other chronic diseases.^21^

Worldwide smoking is still seen mainly as a male problem. Overall prevalence was about four times higher in men than women globally (48% versus 12%) and in the South East Asia region the World Health Organization observed a high prevalence of smoking in men ranging from 25% to 60%.^22^ The smoking problem in Bangladesh is masked by the very significant sex difference because relatively few women smoke. In the present study the prevalence of smoking in women in Bangladesh was low (2.3% current, 3.8% past and 0.7% occasional smoking) while for males the prevalence was 42.2% current, 13.1% past and 9.1% occasional smoking. This significant sex difference in smoking is supported by other studies from Bangladesh in both urban and rural areas^11^ and the BBS reported 10 times higher prevalence in males than females (43.8% in males and 4.6 in females in 1996^9^ and 41% in males and 4.0 in females in 1999^10^). In most countries, male smoking prevalence is higher than female smoking prevalence—for example, in Tanzania (33.3% males and 2% females)^23^ and even in South Africa, where women commonly smoke (20.6%), the male prevalence is still more than double (48%)^24^ while in Europe the ratio is 1.75:1.^25^

Cultural disapproval prohibits women from smoking in Bangladesh.^26^ Because of the social non-acceptance there might be some female under-reporting. Bush *et al* who studied migrant South Asian women living in London reported that the data on smoking might be underestimates (they found about 4% of Bangladeshi, 1% of Indian and 2% of Pakistani women smoked). They reported that a focus group discussion with 18–29-year-old Bangladeshi women indicated “more women are starting to smoke; only they are hidden whilst the men are very open about it. Women smoke in the bedroom with a locked door”.^26^

Although currently women smoke less, there is concern that the numbers may increase as social norms, beliefs, values and taboos are diluted by Western influences,^27^ and the spending power of women increases, so that cigarettes become more affordable.^28^

This study also found higher bidi usage in female smokers, which is in keeping with the BBS which found that women were six times more likely to smoke bidis than cigarettes.^9^ Women in Bihar, parts of Punjab and Haryana states of India smoke bidis and hukkah, mostly.^29^ In New Delhi, India, the prevalences of bidi usage among men and women aged 25–64 years were 21.3% and 6.7% respectively, while for cigarettes they were 23.7% and 0.03%, respectively.^21^

Women in this study smoked, on average, fewer sticks (5.8) than males (9.6). The study by Rahman *et al*^11^ concluded that except for the upper urban socioeconomic class, women in urban and rural areas smoked fewer cigarettes but the conclusion was based on only a few female smokers. In addition, the percentage of heavy (>20 sticks/day) and moderate (10–20 sticks/day) smokers was significantly higher in males than females in the current study (14.1% and 47.4% versus 4.8% and 8.6% in males and females, respectively). No other Bangladeshi or regional data reported on levels of smoking.

The prevalence of chewing tobacco in males and females in the current study was 19.4% and 21.6%, respectively, which is much lower than rates in adult Bangladeshi slum dwellers (35.8% versus 30.7%, respectively)^30^ but similar to a general Bangladeshi male sample which found that 20.5% chewed tobacco.^31^ In contrast to smoking, rates of oral tobacco use were very high in Bangladesh.^9^ Traditionally there is no disapproval of women using smokeless tobacco and most Bangladeshi women who use tobacco, use it in a smokeless form.^4^ In Karachi, Pakistan 21% of men and 12% of women chewed tobacco.^4^ The prevalences of smokeless tobacco in men and women in India were 28.1% and 12% but prevalences varied considerably between states; in Nagpur, 30.8% and 12.6%,^19^ in Kerala 26.8% and 26.4%^4^ while in Mumbai 57.3% and 55.6%,^20^ for males and females, respectively. No significant sex differences were observed in the prevalence of gul use in the current study.

Although the smoking rates were low in Bangladeshi females they were not free of tobacco hazards since women smokers commonly used bidi, and chewing tobacco was more common in females. Both offer higher risk of developing cancer and heart diseases.

Overall, the current smoking in the rural area was about 2.8% higher than in the urban area although the difference was not significant after controlling for other sociodemographic variables. But urban residents were more likely to be occasional smokers (OR 14.92) and less likely to be past smokers (OR 0.51). Rahman *et al* found a 6% higher smoking rate in rural than urban areas (25.6% versus 19.6%)^11^ and the BBS surveys (1996,^9^ 1999^10^) also found higher rates in rural areas in both males and females. In the current study no locality difference was found in smoking status among the male respondents while female respondents smoked more commonly in the rural (4.2%) than in the urban area (0.3%).

In terms of type of smoking, cigarettes were used by more than 90% of urban and 55% of rural smokers in the current study, but there seems to be considerable variation between Bangladeshi studies. For example, Rahman *et al* found that all smokers used cigarettes,^11^ while in a lower socioeconomic urban male study 23.7% and 31.0% were bidi and cigarette smokers, respectively^21^ while in rural Bangladesh, 57% of smokers used bidi.^21^ On average, rural residents in the current study smoked two sticks more per day than urban residents (11 versus 8 sticks/day) which is in keeping with the results of Rahman *et al*.^11^

After adjusting for the other sociodemographic variables, urban residents were less likely to chew tobacco while they were 3.6 times more likely to use gul than rural residents which is consistent with the findings of Rahman *et al*.^11^ No locality differences in chewing tobacco were found in either a male Bangladeshi study^31^ or in an Indian study involving both sexes.^32^ Rural residents deserve more attention in tobacco control programmes since they usually smoke bidis and at a greater frequency. Moreover, the higher prevalence of chewing tobacco in the rural area provides additional health hazards.
